# Analyzing the Underlying Structure of Online Teaching During the COVID-19 Pandemic Period: An Empirical Investigation of Issues of Students

**DOI:** 10.3389/fpsyg.2021.605138

**Published:** 2021-04-15

**Authors:** Muhammad Zeeshan Shaukat, Abdul Aziz Khan Niazi, Tehmina Fiaz Qazi, Abdul Basit

**Affiliations:** ^1^Faculty of Management Studies, University of Central Punjab, Lahore, Pakistan; ^2^Institute of Business and Management, University of Engineering and Technology, Lahore, Pakistan; ^3^Hailey College of Banking and Finance, University of the Punjab, Lahore, Pakistan; ^4^Lahore Institute of Science and Technology, Lahore, Pakistan

**Keywords:** COVID-19 pandemic, ISM, MICMAC, online classes, Pakistan, student issues

## Abstract

The aim of the study is to reveal the underlying structure of issues of university students taking online classes during the COVID-19 pandemic period. The overall design of the study includes a review of contemporary literature and field survey for data collection and analysis. Discourse of literature coupled with expert opinion has been employed for identification of issues. Interpretive Structural Modeling (ISM) is used for the determination of intra-issue relationships and analyzing the underlying structure. Cross impact matrix multiplication applied to classification (MICMAC analysis) is used as a technique for classifying issues on the basis of driving–dependence power. Results of the literature show that there are 21 major issues faced by the students taking online classes. ISM shows that lack of institutional guidelines, lack of regulators’ guidelines, stress of pandemic situation, and abrupt (not planned) start of online classes are the most critical issues. MICMAC analysis reveals that there is no autonomous issue, 4 (i.e., connectivity issue, shy to use technology, lack of institutional guidelines, and stress of pandemic situation) are independent, 6 other issues are dependent, and the remaining 11 are linking. This is a valuable study having practical implications for regulators, students, parents, and society to understand the current problem. It is an original attempt that contributes toward literature in the form of a structural model and a diagram of classification of issues.

## Introduction

During the month of December 2019, the outbreak of a viral pandemic in the city of Wuhan, China, threatened the whole world (Huang et al., 2020). Its quick proliferation and spread endangered humanity. Alarmed at this pandemic’s quick spread, governments decided to close all crowded places, including but not limited to educational institutions. Businesses the world over were temporarily almost discontinued and closed. Lockdowns were announced all over the world; educational institutions were most probably the first hit by the lockdowns and closures. The educational institutions straightforwardly went under forced closure for a couple of months. Since the actions of lockdowns and closures have been adapted by the governments as a solution to contain the proliferation and spread of the pandemic, universities had no option but to embark on online classes following the philosophy of “suspending classes without stopping learning” (Zhang et al., 2020). Although online classes are not a new concept and already has a long history (Larreamendy-Joerns and Leinhardt, 2006), embarking on online classes massively and unpreparedly in panic is a new experience that is not free of problems. Students, teachers, and universities are confronted with lots of unprecedented issues exacerbated by unpreparedness. Literature on the topic of online classes is rich, but it does not account for the issues that are peculiar to the COVID-19 pandemic period and the current unpreparedness of the stakeholders. It is imperative to investigate the issue of online education, particularly from the perspective of those who are ultimately affected by it (Zhang et al., 2020). Therefore, this study has the aim of identifying the issues faced by students in online classes during the COVID-19 pandemic period. There are hardly any studies addressing the issues faced by students during online classes, especially regarding the pandemic situation where both students and institutions were not prepared for the situation. The study identifies the issues faced by the students and addresses policy makers to use the results for the improvement of the quality of education during not only COVID-19 but in the future as well. The aim of the study is also extended to determine the relationships among these issues, to impose hierarchy on them, and to classify them on the basis of driving–dependence power. This study will also discuss mathematically derived results qua reality. Number of methodologies (e.g., TOPSIS, SWARA, GRA, VIKOR, SEM, etc.) was considered to achieve these objectives, but the most appropriate methodology found from the literature is Interpretive Structural Modeling (ISM) (Warfield, 1973, 1974; Sushil, 2017). This methodology has been used over a period of time in numerous studies concerning a multitude of problems like the one in hand. Using ISM in combination with other methodologies (like AHP, ANP, IRP, SEM, PCA, MICMAC, DEMATEL, Fuzzy-MICMAC, and Kappa Analysis) is also common and meaningful. This study uses the combination of ISM and MICMAC analysis in order to substantiate results. The rest of the paper is arranged as follows: *Literature Review*, *Solution Methodology*, *Results and Discussion*, and *Conclusion*.

## Literature Review

Before investigation of the issue at hand, it is vital to survey the influx of current literature concerning online education. Review of literature has been reported in two parts, i.e., representation of literature in general and localizing the issues of online classes from within the literature in particular. Crawford et al. (2020) documented 20 countries that embarked on online classes for higher education during the COVID-19 pandemic period. Fischer et al. (2020) gathered data from 23,610 students of a public sector university and found that students’ grades point average were slightly higher in face-to-face courses as compared to online courses. Kang et al. (2020) addressed the issue of online learning and revealed that every source domain has an effect on target domain task. In the context milieu of the COVID-19 epidemic, Wang et al. (2020) advocated that the teaching system must be flexible and modifiable by way of research, practical, and discussion using information technology. During the novel COVID-19 outbreak, an inimitable concept of “autonomous learning” has been opted by an elementary school in Nigbo city, Zhejiang province, China, to promote independent distance learning and to provide online academic counseling to the students (Xie and Yang, 2020). Andersson (2019) expressed that online distance learning has changed the dynamics of the learning system by providing flexibility, mobility, transition, and campus independence. Luo and Kalman (2018) argued that summary videos help students in learning, reinforcing, and disseminating knowledge; engage students socially, emotionally, and rationally; and intrinsically motivate them in online classes. Baranik et al. (2017) asserted that mentoring relationship needs to be incorporated in online classes in order to combat psychological needs of the students and make online classes successful. Galyon et al. (2016) studied the role of class participation and group cohesion in an online hybrid course and traditional in-class setting and found that class participation is identically high in both formats, whereas group cohesion is reported very low in online classes. Song et al. (2016) revealed that teacher self-disclosure plays a pertinent role in the development of satisfied student–teacher relationship in online classes that resultantly increases class satisfaction and knowledge gain. Bourelle et al. (2015) found that incorporation of instructional assistants in online classes provides students feedback on their write-up, active interaction with instructors, and personalized attention throughout the online classes. Oswal and Meloncon (2014) urged that it is important to devise strategies for easy accessibility of online courses. Gray (2013) argued that there is an increase in enrollment of students in online classes of post-secondary educational institutions in United States. Baxter (2012) pointed out critical factors regarding students’ progress in distance learning in terms of pliability and motivation to remain with course. Hartnett et al. (2011) and Macintyre and Macdonald (2011) argued that the success of distance learning largely depends on motivation of students, geographical distance, situational conditions, and individual circumstances. Qiu et al. (2012) examined the effect of class size on students’ performance in online classes and asserted that students are more likely to experience overload. It also argued that it is beneficial to have a small class size (i.e., 13–15 students) for effective and collaborative discussion to improve students’ performance. Jeong (2007) stated that instant messaging is a unique and useful medium of communication between teacher and student in online classes in higher education. Tallent-Runnels et al. (2006) conducted a systematic review of literature on teaching courses online and found that the students who have prior knowledge and training of computer are more satisfied with online class.

### Localizing the Issues of Online Classes From Within Contemporary Studies

Alghamdi et al. (2020) argued that a multi-tasking approach in online classes has an adverse effect on the academic performance of students. Zhang et al. (2020) highlighted the number of problems of online classes and proposed some suggestions to the Chinese government to tackle the problems encountered during the COVID-19 outbreak, including equipping students and teachers with home-based learning/teaching equipment, devising a massive strategic plan for online education, developing an educational information superhighway, organizing online training, and assisting academic research to promote online education. Bawa (2016) explored issues in lack of retention of students in online classes and proclaimed that personal preferences, workload misconception, expectations, lack of skill of using technology, and cognitive challenges are critical issues that impede the effectiveness of online classes. El-Magboub et al. (2016) proclaimed that conducting online classes may lessen participation and interaction, which requires active facilitator–student interactions and a participation approach that differs from in-class/face-to-face discussions. Kranzow (2013) argued that the role of faculty leadership is vital in structuring courses/design curriculum in online education, which has a significant impact on student satisfaction and motivation. In connection to this, faculty must be trained to teach online classes (Shahdad and Shirazin, 2012). Willging and Johnson (2009) and Aragon and Johnson (2008) argued that students’ decision to completion/non-completion and dropout is unique to each student in online classes. Bolliger (2004) identified three key issues of online education: (i) instructor capability (including preparation, teaching methodology, feedback, professionalism, knowledge, and communication), (ii) low interactiveness, and (iii) technology issues (connectivity appropriateness, etc.). Zhang et al. (2020) asserted that online classes have several issues, viz, this public emergency management led mechanism is relatively trivial as yet, there is persistent information gap among stakeholders, it is difficult to address disparity in teacher quality and educational resources, there is lack of mature detailed plans for massive online classes in emergency time, it is on teachers’ shoulders with few guidelines from institutions and regulators to solve practical problems during the conduct of online classes, there is a difference between environment of learning at home and on-campus, internet is a less effective platform for teacher–student interface, and long-term online teaching has negative effects on the mental and physical health of students. The literature is rich in proposing methodologies for identifying different elements of phenomena under study. The commonly suggested methods are as follows: literature review (Azevedo et al., 2013; Kumar et al., 2013; Li et al., 2019), expert opinion (Li et al., 2019), case study method (Li et al., 2019), Delphi method (Bhosale and Kant, 2016), exploratory factor analysis (Li and Yang, 2014), meta-analysis (Lohaus and Habermann, 2019), idea engineering workshop and brainstorming session (Kumar et al., 2013), interview content analysis (Xiao, 2018), empirical evidence provided by different studies, and literature review based on purposive sampling from literature (Azevedo et al., 2013). This study has adopted literature review in order to identify the issues and expert opinion to validate the issues. In a nutshell, a list of the issues faced by students taking online classes during this pandemic period has been finalized (Table 1).

**TABLE 1:** Issues faced by university students taking online classes during the COVID-19 pandemic period.

Code	Issue	Description	Literature support
1	Adaptability struggle	Students struggle to adapt to the processes of online classes for an intermediary period.	Hartnett et al., 2011
2	Self-discipline difficult	It is difficult for a student to impose self-discipline and become serious.	Alghamdi et al., 2020
3	Boring	Formal on-campus classes are interactive and interesting but online classes are boring for students.	Xie and Yang, 2020
4	Lack of proficiency in IT	Certain level of proficiency in IT applications is required to participate in online classes and mostly students do not have that proficiency.	Zhang et al., 2020
5	Connectivity issues	There is extreme load on networks during this pandemic period; therefore, there are serious issues of connectivity being faced by many students.	Macintyre and Macdonald, 2011; Zhang et al., 2020
6	Not-fit in all subjects	Online classes are possible for certain subjects; it is not suitable for every subject.	Alghamdi et al., 2020
7	Casual settings distraction	At home, there is an atmosphere of casual setting and taking online classes is subject to continuous interruption.	Zhang et al., 2020
8	Lack of practice	Since students are lacking in practice, there are some unforeseen issues.	Wang et al., 2020
9	Teachers’ IT proficiency	The teachers teaching really well on campus might not be that proficient in online lecturing/recording and making slides, etc.	Kranzow, 2013
10	Market acceptability issue	Students are confused about acceptability of these courses in market.	Willging and Johnson, 2009
11	Assessment confusion	How will students be assessed for learning in online classes? Criteria are not clear.	Alghamdi et al., 2020
12	More-work little-focus	Teachers disseminate a lot of information online without realizing practical problems with students; students, despite putting a lot of effort, learn little.	Luo and Kalman, 2018
13	No class participation	At the university level, students learn a lot through class participation and interaction with their fellow students, which is not possible in online classes.	El-Magboub et al., 2016
14	Seems non-realistic	After a long on-campus classes’ journey, online classes seem to be non-realistic and temporary phenomena.	Baxter, 2012
15	Unavailability of equipment	It is not necessary that every student has a laptop/computer system/smartphone. The complete lockdown of markets makes it difficult to make equipment available.	Wang et al., 2020
16	Unavailability of internet	There are certain areas where there is no internet facility, re-charge facility, or even electricity.	Wang et al., 2020
17	Shy to use technology	Some students have an unknown fear of using technology, so they are afraid of using technology and are not able to properly benefit from online classes.	El-Magboub et al., 2016
18	Abrupt not planned	Clearly, this pandemic outbreak is sudden and online classes are abruptly started to cover the pandemic period; students believe that there is lack of planning.	Zhang et al., 2020
19	Lack of institutional guideline	Since it is all abrupt, either there are no guidelines or there are uncoordinated and inconsistent guidelines from institutes/departments/universities.	Yang et al., 2020; Zhang et al., 2020
20	Lack of regulators’ guideline	Since it is all abrupt, either there are no guidelines or there are uncoordinated and inconsistent guidelines from regulatory bodies.	Hartnett et al., 2011; Zhang et al., 2020
21	Stress of pandemic situation	At present, students, like all other members of society, are stressed due to the COVID-19 pandemic situation; hence, their focus is not on studying.	Yang et al., 2020

## Solution Methodology

This study follows a post-positivist research philosophy and qualitative paradigm of research. The overall design of research is envisaged on discourse of literature review regarding the phenomenon under study and collection of primary data by way of field survey and analysis. The population under study involves university students affected by lockdowns and forced foreclosures pressed to take classes online. Primary data have been collected from class representatives of students (so taking the classes) using a non-random purposive sampling technique (Ranjbar et al., 2012). The methodologies used for investigation and analysis of phenomenon are ISM and MICMAC. These methodologies are based on permutations of binary metrics using elementary concepts of Boolean algebra, set theory, and graph theory. A matrix-type questionnaire commensurate to structural methodologies has been used to elicit the data (Alawamleh and Popplewell, 2011). The respondents were first approached over the telephone, and subsequently, questionnaires were sent via emails. The authors (being university teachers) used the privileged information provided by the universities regarding class representative students in order to collect these data. Keeping in mind the essence of ISM, the data were collected from 42 students’ class representatives recruited for the study on the basis of predetermined criteria like a panel of experts. The data have been collected from class representatives (i.e., male and female) of post-graduate classes from the 22–35 year age group.

### Panel of Experts

The essence of the methodology demands the collection of data from a panel of experts having complete knowledge, expertise, and experience of the issue under investigation (Shen et al., 2016). This way of data elicitation is ideal when statistical data are non-existing or expensive or not possible to collect. The panel of experts outperform other methodologies in eliciting intra-factor relations. There are several methods to elicit the data from respondents, viz, Delphi method, brainstorming session, discussion session, nominal group technique, repertory-grid interview technique, laddering interview, problem solving group session, in-depth discussion, one-to-one face-to-face in-depth interview, triadic sorting task approach, approval voting on alternatives for every pair of relations through software, elect alternatives for every pair of relations, workshops of idea engineering or idea generation method with small group exercise, and matrix-type survey questionnaire. The present study is an investigation of issues of students who are bound to take online classes during the COVID-19 pandemic period; therefore, the students are the best respondents. The panel of experts or respondent focus group has, therefore, been constituted from within the students on the basis of predetermined criteria. The criteria for recruitment on the panel of experts/respondent focus group include the following: 4 years university study (approximately), acumen of the student to appreciate the research, familiarity with multi-criteria decision techniques, taking online classes during this COVID-19 pandemic period, officially holding position of class representative, and willing to participate in study. Using aforementioned privileged information, the authors identified 50 class-representative boys and girls from within the leading universities of Pakistan. Forty-five students consented to participate in this research activity. The students were briefed about the background of the study via the telephone (Li and Yang, 2014; Shen et al., 2016). The list of issues with description was sent to recruited students before preparation of questionnaire. They had the option to include, exclude, or merge the issues. The list was finalized on the basis of majority rule (Li et al., 2019). The questionnaire was then prepared and mailed. They were also briefed about the rules of completing the questionnaire. A total of 45 questionnaires were mailed, out of which 42 responses were timely received. During the screening of the questionnaires, three were found to be inappropriately filled. A total of 39 responses have been used for this study. This size of respondents’ panel is appropriate for the study (Clayton, 1997; Khan and Khan, 2013). The data collected by way of a matrix-type questionnaire have been aggregated by calculating the mode value for each pair of relationship. Panels of experts were also referred back for evaluation of ISM model for inconsistencies.

### Interpretive Structural Modeling

It is a visible, well-defined, graphical model representation using reachability and transitive inferences through matrix transformation. It transforms unclear and poorly articulated mental models of systems into visible, well-defined models useful for many purposes (Sushil, 2017). It is workable with as few as 5 and as many as more than 90 elements (Sushil, 2017; Li et al., 2019). It is applied in a wide variety of situations. It has the competence to develop a primary model. This technique determines different levels of design characteristics: “drivers” (the lower level), “facilitators” (middle level), and “dependents” (top level). It does not use *a priori* theoretical framework and raises awareness in academics and practitioners by providing information during challenging situations. It proceeds stepwise (Warfield, 1973; Thakkar et al., 2008; Attri et al., 2013) viz identifying elements and establishing the contextual relationship between them, development of the Structural Self-Interaction Matrix, development of the reachability matrix, partitioning the reachability matrix, development of the conical matrix, development of a digraph, development of the ISM model, and checking models for conceptual inconsistencies.

The issues concerning the phenomenon under investigation have already been identified (Table 1) as the first step of ISM. Data are collected by way of field survey from 39 respondents. Their mental models about contextual relationships among the issues are captured on $\frac{n\left(n-1\right)}{2}$ matrix-type questionnaires. Using the relationship logic of “leads to,” rules followed by respondents to determine every paired relation are as follows: *V*: if row leads to column, *A*: if column leads to row, *X*: if row and column are two-way related, and *O*: if row and column are not related.

#### Development of the Structural Self-Interaction Matrix (SSIM)

The data collected from respondents are aggregated using the principle of most frequent value (modal value) for developing SSIM (Table 2).

**TABLE 2:**
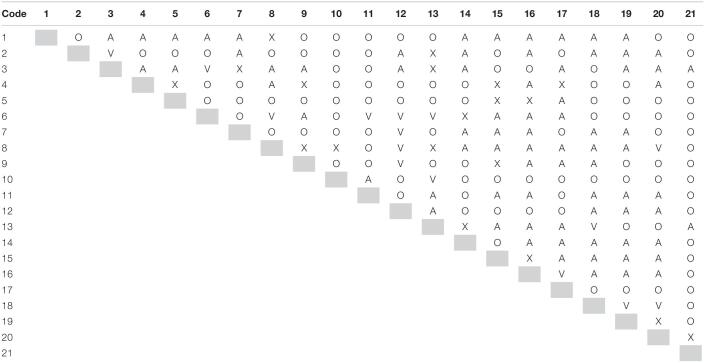
Structural Self-Interaction Matrix (SSIM).

SSIM is then converted into an initial reachability matrix by binary coding of the relationships denoted by way of *VAXO* symbols.

#### Constructing an Initial Reachability Matrix

Initial reachability matrix (Table 3) is created from SSIM using the following rules:

• If *ij* entry in SSIM is *V*, then *ij* entry in the initial reachability matrix is 1 and *ji* entry in initial reachability is 0.
• If *ij* entry in SSIM is *A*, then *ij* entry in the initial reachability matrix is 0 and *ji* entry in initial reachability is 1.
• If *ij* entry in SSIM is *X*, then *ij* entry in the initial reachability matrix is 1 and *ji* entry in initial reachability is 1.
• If *ij* entry in SSIM is *O*, then *ij* entry in the initial reachability matrix is 0 and *ji* entry in initial reachability is 0.

**TABLE 3:**
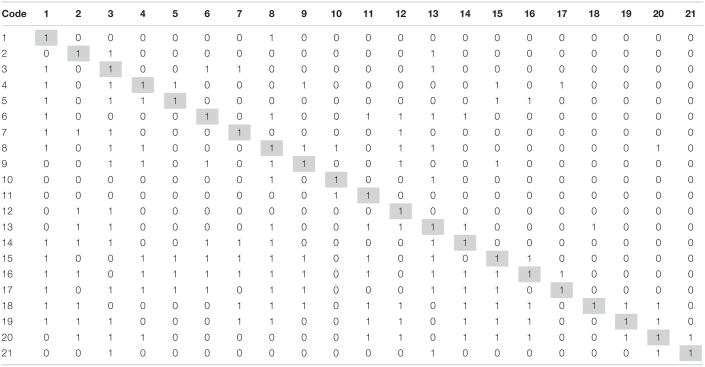
Initial reachability matrix.

In the initial reachability matrix, direct relationships among factors are indicated by 1s. However, it is quite possible that there may be indirect relationship between the factors, that indirect relationship is termed transitive relation, e.g., *a* leads to *b* and *b* leads to *c*; therefore, it is logical to say that *a* leads to *c* (transitive or indirect relationship).

#### Developing a Fully Transitive Reachability Matrix

Before embarking on partitioning, it is necessary to check out the initial reachability matrix for transitivity. In this way, a fully transitive matrix is constructed (Table 4). It can be observed that some of the 0s in the initial reachability matrix have been converted into *1*^∗^ while developing a final reachability matrix. The symbol *1*^∗^ indicates transitive relationship.

**TABLE 4:**
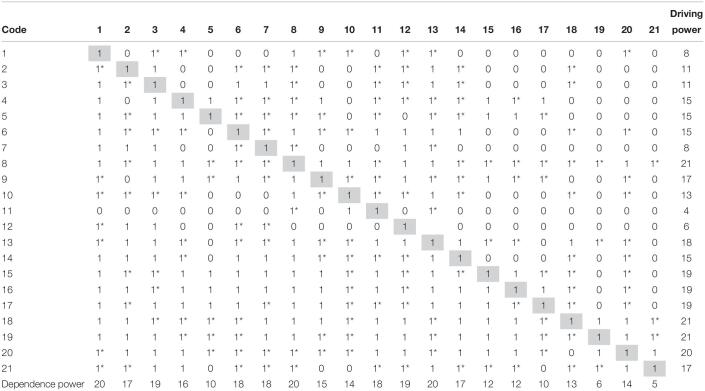
Final reachability matrix.

The final reachability matrix can be further manipulated for hierarchies in order to reveal the structural model behind this binary matrix. The last column of the fully transitive reachability matrix is the driving power of each factor, which is computed by counting 1s in the respective rows, whereas the last row of the matrix is the dependence power of every factor that has been computed by counting 1s in the respective columns. Driving and dependence power have been used subsequently for constructing a MICMAC diagram.

#### Level Partitioning

In order to determine the levels of the ISM model, a partitioning method has been employed. It uses elementary concepts of set theory to manipulate the fully transitive reachability matrix (Table 4). The reachability set, antecedent set, and intersection set of each factor is determined. The reachability set consists of the factor itself and the factors to which it leads, the antecedent set consists of the factor itself and the factors that lead to it, whereas the intersection set consists of the common factors of reachability and antecedent. While during the first iteration of level partitioning, the factor, against which the reachability set is identical to the corresponding intersection set, that factor will occupy highest level (Level I) in ISM model. Once the level of any factor is determined, such factor is eliminated from further iterations. Eliminating the factor in this manner leads to at least one more factor having similar reachability and intersection sets; therefore, the factors occupying the next level (i.e., *Level II*) are identified. This process is iterated till the levels of all factors are determined (Supplementary Tables 1–8).

#### Development of the Conical Matrix and Digraph

On the basis of the levels determined by way of partitioning, a conical matrix is developed, and from the conical matrix, a diagraph emerged. Being optional in the ISM procedure, the conical matrix and diagraph have not been reported (Sushil, 2012).

#### Development of the ISM Model

**FIGURE 1:**
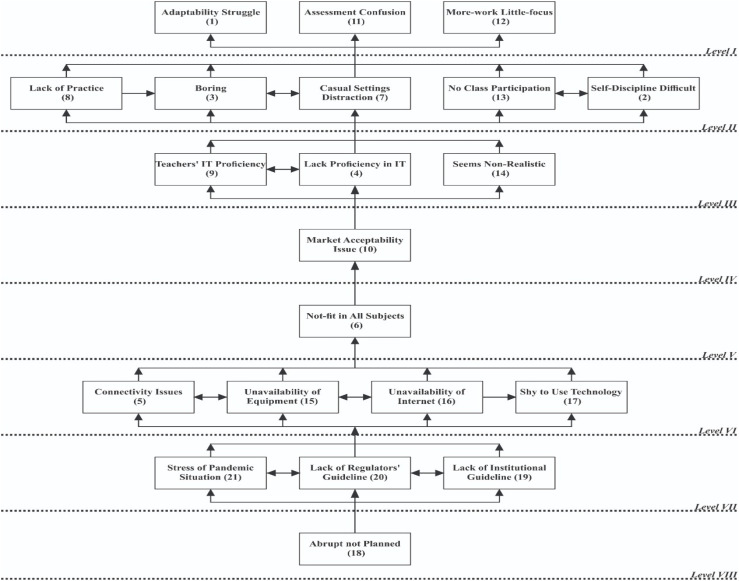
Interpretive Structural Modeling (ISM).

Following the classical procedure devised by Warfield (1973), the ISM model has been constructed as Figure 1.

Close observation of Figure 1 (ISM Model) reveals that (i) 1, 11, 12 and 2, 3, 7, 8, 13 occupy the top part of the model, i.e., *Levels I and II*, respectively, and hence are less critical issues; (ii) 5, 15, 16, 17, 19, 20, 21, and 18 occupy the bottom part of the model (*Levels VII and VIII*) and therefore are the most critical issues; and (iii) 4, 9, 14, 10, and 6 occupy the middle part of the model (*Levels III, IV, V, and VI*) and are mediating issues, whereas 19, 20, 21, and 18 are the key issues. Level of ISM Model are marked in Roman numbers and italicized just to distinguish them from within the running text.

#### Check for Conceptual Inconsistency

Following the recommendations of Raeesi et al. (2013) and Vasanthakumar et al. (2016), the respondents were approached again and were asked to check the conceptual inconsistencies (if any). The model was reviewed and found to be in order and consistent.

### MICMAC Analysis

It is a structural methodology introduced by Godet (1986). It is commonly used as a complementary method to ISM. It uses the data of the fully transitive reachability matrix. Following the data-centered approach, a driving–dependence diagram is drawn that classifies the issues into four clusters, namely, autonomous, independent, dependent, and linkage.

**FIGURE 2:**
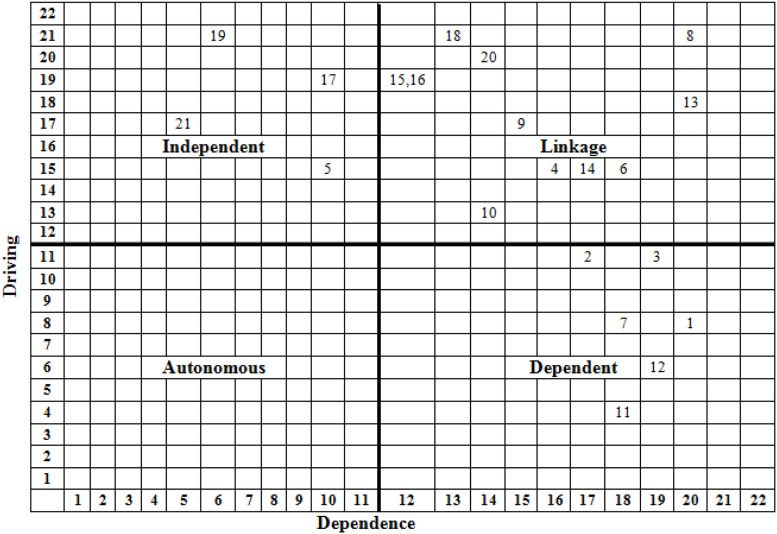
Driving–dependence diagram.

Close observation of Figure 2 (Driving–Dependence Diagram) reveals that (i) 1, 2, 3, 7, 11, and 12 are dependent issues; (ii) 5, 17, 19, and 21 are independent factors; (iii) 4, 6, 8, 9, 10, 13, 14, 15, 16, 18, and 20 are linkage; and (iv) there is no autonomous issue as such.

## Results and Discussion

### Results

Education is the backbone of economies particularly in this regime of knowledge economies. Higher education has gained great importance, but the current COVID-19 pandemic situation badly shattered the system of university education. The prolonged lockdown of educational institutions has raised many new questions like those under investigation in this study. It has become imperative to investigate the issue from the viewpoint of students. It is a vital problem of students who are abruptly forced to take online classes without having any other option. This study aims to identify the issues of students taking online classes during the COVID-19 pandemic period, impose hierarchy on them, and classify them to make some sense of the system for stakeholders. Issues have been identified through discourse of literature, and ISM and MICMAC analysis have been employed as techniques of investigation. The results of these techniques provide deeper understanding to the stakeholders. The results of literature discourse revealed that there are 21 critical issues presently (i.e., during the COVID-19 pandemic period) being faced by university students regarding online classes (Table 1). Results of ISM show that:

i adaptability struggle (1), assessment confusion (11), more-work little-focus (12), self-discipline difficult (2), boring (3), casual settings distraction (7), lack of practice (8), and no class participation (13) occupy the top part of the model; therefore, they are the least critical issues.
ii connectivity issues (5), unavailability of equipment (15), unavailability of internet (16), shy to use technology (17), lack of institutional guideline (19), lack of regulators’ guideline (20), stress of pandemic situation (21), and abrupt not planned (18) occupy the bottom part of the model and therefore are the most critical issues.
iii lack of proficiency in IT (4), teachers’ IT proficiency (9), seems non-realistic (14), market acceptability issue (10), and not-fit in all subjects (6) occupy the middle part of the model and play role of mediators.
iv lack of institutional guideline (19), lack of regulators’ guideline (20), stress of pandemic situation (21), and abrupt not planned (18) are key issues.

The results of MICMAC show that:

i There is no issue falling in the *autonomous cluster*. The factors that fall in the autonomous cluster have a weak driving and a weak dependence power. They are separated from the model but have few powerful links. In fact, they do not have much impact on the system. Non-existence of autonomous factors means all factors identified and studied are relevant and play an important role, and practitioners should pay attention to all issues. In this study, there is no autonomy; hence, all issues identified are relevant and need the attention of stakeholders.
ii adaptability struggle (1), self-discipline difficult (2), boring (3), casual settings distraction (7), assessment confusion (11), and more-work little-focus (12) fall in the *dependent cluster*. The factors that fall in this cluster have a weak driving but a strong dependence power. They resultantly depend on others and they need extra care. There are some factors having high dependence that may also fall in linkage because of their having high driving as well.
iii lack proficiency in IT (4), not-fit in all subjects (6), lack of practice (8), teachers’ IT proficiency (9), market acceptability issue (10), no class participation (13), seems non-realistic (14), unavailability of equipment (15), unavailability of internet (16), abrupt not planned (18), and lack of regulators’ guideline fall in the *linkage cluster*. The factors that fall in this cluster have a strong driving and a strong dependence power. They are unbalanced, agile, and ambivalent. Any action on them affects others and, as a feedback, affects themselves. Clustering of more factors as linkage means that the phenomenon is in its infancy and stakeholders are struggling to make some sense.
iv connectivity issues (5), shy to use technology (17), lack of institutional guideline (19), and stress of pandemic situation (21) fall in the *independent cluster*. The factors that fall in this cluster have a high driving but a low dependence power. However, there may be some factors that have a high driving and, at the same time, a high dependence power and may instead fall in linkage. Independent factors are key factors; therefore, great care is needed to handle them. Stakeholders should therefore give priority to handle these factors.

Aforementioned results have been summarized and juxtaposed (Table 5) in order to make it clear and simple.

**TABLE 5:** Juxtaposed results of literature, MICMAC, and ISM.

Result of literature review		Results of MICMAC analysis				Results of ISM	Comments
Code	Issue	Driving	Dependence	Effectiveness	Cluster	Level	
1	Adaptability struggle	9	20	−11	Dependent	*I*	
2	Self-discipline difficult	11	17	−6	Dependent	*II*	
3	Boring	11	20	−9	Dependent	*II*	
4	Lack proficiency in IT	15	16	−1	Linkage	*III*	
5	Connectivity issues	15	10	4	Independent	*VI*	
6	Not-fit in All subjects	15	18	3	Linkage	*V*	
7	Casual settings distraction	8	18	−10	Dependent	*II*	
8	Lack of practice	21	20	1	Linkage	*II*	
9	Teachers’ IT proficiency	17	15	2	Linkage	*III*	
10	Market acceptability issue	13	14	−1	Linkage	*IV*	
11	Assessment confusion	4	18	−14	Dependent	*I*	
12	More-work little-focus	6	19	−13	Dependent	I	
13	No class participation	18	20	−2	Linkage	*II*	
14	Seems non-realistic	15	17	−2	Linkage	*III*	
15	Unavailability of equipment	19	12	7	Linkage	*VI*	
16	Unavailability of internet	19	12	7	Linkage	*VI*	
17	Shy to use technology	19	10	9	Independent	*VI*	
18	Abrupt not planned	21	13	8	Linkage	*VIII*	Key factors
19	Lack of institutional guideline	21	6	15	Independent	*VII*	Key factors
20	Lack of regulators’ guideline	20	14	7	Linkage	*VII*	Key factors
21	Stress of pandemic situation	17	5	12	Independent	*VII*	Key factors

### Discussion

The main objective of study is to expound the issues of the students concerning online classes particularly during this COVID-19 pandemic period and impose hierarchy on the intra-factor relationships to unearth the underlying structure of the phenomenon. In order to achieve this objective, the class representatives of students were approached for data collection and then a procedure of two structural methodologies was applied on the data to reveal the underlying mental model of issues of online classes being faced by students, and ultimately, the model has been drawn. There are a couple of recent studies on the issue; therefore, a contrast of the study in hand with contemporary studies has been drawn (Table 6). The current study is different from contemporary studies on many counts. It uses a different qualitative approach of ISM that is appropriate for making some sense of the present challenging situation. It also accounts for the full range of issues instead of taking into account two or three variables. It is also different since it examines the perspective of those who are affected, which has not previously been investigated.

**TABLE 6:** Contrasting results of the study with contemporary studies.

Sr.	Study	Focus	Country	No. of factors	Key factors	Methodology
1	Current study	Issues being faced by students regarding online classes during the COVID-19 pandemic period.	Pakistan	21	Abrupt and unplanned start of classes, lack of institutional guidelines, lack of regulators’ guidelines, and stress of pandemic situation.	ISM
2	Wang et al. (2020)	Chinese universities’ contributions to emergency risk management.	China	5	Problems of alumni’s economic development difficulties, risk of infection to health workers, infection of teachers and students, and the unsatisfactory application of IT. There are proposed solutions for issues on medical security, emergency research, professional assistance, positive communication, and hierarchical information-based teaching.	Theoretical Editorial
3	Xie and Yang (2020)	Autonomous learning of elementary students at home during the COVID-19 epidemic.	China	–	Prepare targeted learning materials to promote students’ autonomous learning.	Theoretical Report
4	Zhang et al. (2020)	Suspending classes without stopping learning in schools.	China	5	Weakness of online teaching infrastructure, inexperienced teachers, information gap, complex environment at home.	Theoretical Editorial
5	Kang et al. (2020)	Online Transfer Learning (OnLT) with multiple source domains.	China	–	Online transfer learning	Mathematical algorithm
6	Alghamdi et al. (2020)	Indirect effects of multitasking on academic performance in both face-to-face and online classes.	United States	4	Multitasking in classroom negatively affects students’ performance.	Classical statistical tools to check moderated mediation
7	Aydemir and Ulusu (2020)	Challenges for Ph.D. students in bio-chemistry and molecular biology fields during the COVID-19 pandemic.	Turkey	–	COVID-19 crisis should be converted into an opportunity to learn.	Commentary

The current study is a unique study because it investigates the subject that has not directly been investigated particularly with reference to the COVID-19 pandemic period. The studies conducted regarding education during the COVID-19 pandemic period are theoretical reports or editorials, rather than research studies. The authors could not find an empirical study on the problem under investigation. The only empirical study one can find is Alghamdi et al. (2020), and that too is different in context and does not give any insight into the topic under study. Most studies have been conducted in China, and there is hardly any study for the rest of world on this topic. Most studies did not use scientific methodologies like ISM or MICMAC and are argumentative. The study results would be very helpful for higher education commissions and policy makers for policy making and guiding the educational institutes by considering student issues. By utilizing the results of this study, educational institutes can make more student-oriented policies and run their educational operations more smoothly.

## Conclusion

With the outbreak of the COVID-19 pandemic, pseudo-natural systems have been shattered, inter alia, economic, financial, and social systems, and the educational system was the worst hit. All of a sudden, the lockdown of educational institutions left the stakeholders with no other option but to embark on online classes. It is of great value for stakeholders to investigate the issues concerning this phenomenon at this moment. Unprepared students who abruptly shifted to online classes face a multitude of issues. This is a vital and hot problem that is addressed by this study. The study identified the major issues of students regarding online classes, revealed the underlying structure behind the issues, classified them on the basis of their driving–dependence power, and pointed out the key issues. The study employed discourse of literature review and ISM coupled with MICMAC as a solution methodology. As a result of literature review, 20 issues have been identified (Table 1). Results of ISM show that issues coded as 1, 11, 12 and 2, 3, 7, 8, 13 occupy the top part of the model and hence are less critical, issues coded as 5, 15, 16, 17, 19, 20, 21, and 18 occupy the bottom part of the model and therefore are the most critical, whereas issues coded as 4, 9, 14, 10, and 6 occupy the middle part of the model and are mediating and hence are moderate critical issues. It also reveals that lack of institutional guideline (19), lack of regulators’ guideline (20), stress of pandemic situation (21), and abrupt not planned (18) are key issues. Results of MICMAC show that there is no autonomous issue, 1, 2, 3, 7, 11, and 12 are dependent issues, and 5, 17, 19 and 21 are independent, whereas 4, 6, 8, 9, 10, 13, 14, 15, 16, 18, and 20 are linking. The results of MICMAC are quite aligned with that of ISM, and they also support the idea of key issues (Table 6). The study contributes to existing theories by way of deepening the understanding of the phenomenon in general and provided a refined list of students’ issues (Table 1). Furthermore, the study provided a scientifically developed ISM model (Figure 1) and a mathematically driven “driving–dependence diagram” (Figure 2) to contemporary literature. This qualitative study complements contemporary theoretical and quantitative studies by providing more supplementary information. It also provides novel insights into the phenomenon and a framework for future quantitative studies. The study will help regulators to understand the phenomenon clearly to improve the processes. The results of the study are useful for university management, regulators, students, parents, and the society at large for making decisions regarding pandemic situations. The ISM-based model offers practitioners and policy makers a framework for building awareness on the issues to resolve them as it provides essential information to decision-makers by identifying focal areas. The study also has some limitations. Firstly, data have been collected during the COVID-19 pandemic period using non-probability-based purposive sampling; therefore, generalizability of results should be made with caution and future studies should be conducted to validate the results when this period is over using probability-based larger sampling. Secondly, it is a qualitative study and the model advanced as a result of this study could not be statistically validated; therefore, it is recommended that future studies may validate it using quantitative models like SEM, PCA, AHP, ANP, TOPSIS, GRA, etc. Thirdly, the study has been conducted in Pakistan; similar future studies may also be conducted in other countries. Fourthly, a limited number of issues have been studied and those, too, are validated by students; therefore, it is recommended that, in the future, further issues may be explored with a different set of respondents.

## Data Availability Statement

The raw data supporting the conclusions of this article will be made available by the authors, without undue reservation, to any qualified researcher.

## Ethics Statement

Ethical review and approval was not required for the study on human participants in accordance with the local legislation and institutional requirements. The patients/participants provided their written informed consent to participate in this study.

## Author Contributions

MS conceived the idea and scheme of the study including setting the outset of the study. AN collected and analyzed the data and constructed the model. TQ prepared write-up. AB refined the whole study and flow of thought, and wrote results and conclusion. All authors contributed to the article and approved the submitted version.

## Conflict of Interest

The authors declare that the research was conducted in the absence of any commercial or financial relationships that could be construed as a potential conflict of interest.
