# Supplementary material for: Analyzing the Underlying Structure of Online Teaching During the COVID-19 Pandemic Period: An Empirical Investigation of Issues of Students
Source: Front Psychol. 2021 Apr 15;12:605138. doi: 10.3389/fpsyg.2021.605138 (PMC8084101; doi:10.3389/fpsyg.2021.605138)
Supplement: Supplementary file 1 [file Table_1.docx]

**Supplementary Tables – Iterations**

**Table 1: Iteration 1**

| **Code** | **Reachability Set** | **Antecedent Set** | **Intersection Set** | **Level** |
| --- | --- | --- | --- | --- |
| 1 | 1,3,4,8,9,10,12,13,20 | 1,3,4,8,9,10,12,13,20 | 1,3,4,8,9,10,12,13,20 | *Level I* |
| 2 | 1,2,3,6,7,8,11,12,13,14,18 | 1,2,3,6,7,8,11,12,13,14,18 | 2,3,6,7,8,12,13,14,18 |  |
| 3 | 1,2,3,6,7,8,11,12,13,14,18 | 1,2,3,6,7,8,11,12,13,14,18 | 1,2,3,6,7,8,12,13,14,18 |  |
| 4 | 1,3,4,5,6,7,8,9,11,12,13,14,15,16,17 | 1,3,4,5,6,7,8,9,11,12,13,14,15,16,17 | 1,4,5,6,8,9,13,14,15,16,17 |  |
| 5 | 1,2,3,4,5,6,7,8,9,11,13,14,15,16,17 | 1,2,3,4,5,6,7,8,9,11,13,14,15,16,17 | 4,5,8,9,15,16,17 |  |
| 6 | 1,2,3,4,6,7,8,9,10,11,12,13,14,18,20 | 1,2,3,4,6,7,8,9,10,11,12,13,14,18,20 | 2,3,4,6,7,8,9,12,13,14,18,20 |  |
| 7 | 1,2,3,6,7,8,12,13 | 1,2,3,6,7,8,12,13 | 2,3,6,7,8,12,13 |  |
| 8 | 1,2,3,4,5,6,7,8,9,10,11,12,13,14,15,16,17,18,19,20,21 | 1,2,3,4,5,6,7,8,9,10,11,12,13,14,15,16,17,18,19,20,21 | 1,2,3,4,5,6,7,8,9,10,11,13,14,15,16,17,18,19,20,21 |  |
| 9 | 1,3,4,5,6,7,8,9,10,11,12,13,14,15,16,17,20 | 1,3,4,5,6,7,8,9,10,11,12,13,14,15,16,17,20 | 1,4,5,6,8,9,10,13,14,2,16,17,20 |  |
| 10 | 1,2,3,4,8,9,10,11,12,13,14,18,20 | 1,2,3,4,8,9,10,11,12,13,14,18,20 | 1,8,9,10,11,13,14,18,20 |  |
| 11 | 8,10,11,13 | 8,10,11,13 | 8,10,11,13 | *Level I* |
| 12 | 1,2,3,6,7,12 | 1,2,3,6,7,12 | 1,2,3,6,7,12 | *Level I* |
| 13 | 1,2,3,4,6,7,8,9,10,11,12,13,14,15,16,18,19,20 | 1,2,3,4,6,7,8,9,10,11,12,13,14,15,16,18,19,20 | 1,2,3,4,6,7,8,9,10,11,13,14,15,16,18,19,20 |  |
| 14 | 1,2,3,4,6,7,8,9,10,11,12,13,14,18,20 | 1,2,3,4,6,7,8,9,10,11,12,13,14,18,20 | 2,3,4,6,8,9,10,13,14,18,20 |  |
| 15 | 1,2,3,4,5,6,7,8,9,10,11,12,13,14,15,16,17,18,20 | 1,2,3,4,5,6,7,8,9,10,11,12,13,14,15,16,17,18,20 | 4,5,8,9,13,15,16,17,18,20 |  |
| 16 | 1,2,3,4,5,6,7,8,9,10,11,12,13,14,15,16,17,18,20 | 1,2,3,4,5,6,7,8,9,10,11,12,13,14,15,16,17,18,20 | 4,5,8,9,13,15,16,17,18,20 |  |
| 17 | 1,2,3,4,5,6,7,8,9,10,11,12,13,14,15,16,17,18,20 | 1,2,3,4,5,6,7,8,9,10,11,12,13,14,15,16,17,18,20 | 4,5,8,9,15,16,17,18,20 |  |
| 18 | 1,2,3,4,5,6,7,8,9,10,11,12,13,14,15,16,17,18,19,20,21 | 1,2,3,4,5,6,7,8,9,10,11,12,13,14,15,16,17,18,19,20,21 | 2,3,6,8,10,13,14,15,16,17,18,19,21 |  |
| 19 | 1,2,3,4,5,6,7,8,9,10,11,12,13,14,15,16,17,18,19,20,21 | 1,2,3,4,5,6,7,8,9,10,11,12,13,14,15,16,17,18,19,20,21 | 8,13,18,19,20,21 |  |
| 20 | 1,2,3,4,5,6,7,8,9,10,11,12,13,14,15,16,17,19,20,21 | 1,2,3,4,5,6,7,8,9,10,11,12,13,14,15,16,17,19,20,21 | 1,6,8,9,10,13,14,15,16,17,19,20,21 |  |
| 21 | 1,2,3,4,6,7,8,11,12,13,14,15,16,18,19,20,21 | 1,2,3,4,6,7,8,11,12,13,14,15,16,18,19,20,21 | 8,18,19,20,21 |  |

**Table 2: Iteration 2**

| **Code** | | **Reachability Set** | | **Antecedent Set** | | **Intersection Set** | | **Level** | |
| --- | --- | --- | --- | --- | --- | --- | --- | --- | --- |
| 2 | 2,3,6,7,8,13,14,18 | | 2,3,6,7,8,13,14,18 | | 2,3,6,7,8,13,14,18 | | *Level II* | |  |
| 3 | 2,3,6,7,8,13,14,18 | | 2,3,6,7,8,13,14,18 | | 2,3,6,7,8,13,14,18 | | *Level II* | |  |
| 4 | 3,4,5,6,7,8,9,13,14,15,16,17 | | 3,4,5,6,7,8,9,13,14,15,16,17 | | 4,5,6,8,9,13,14,15,16,17 | |  | |  |
| 5 | 2,3,4,5,6,7,8,9,13,14,15,16,17 | | 2,3,4,5,6,7,8,9,13,14,15,16,17 | | 4,5,8,9,15,16,17 | |  | |  |
| 6 | 2,3,4,6,7,8,9,10,13,14,18,20 | | 2,3,4,6,7,8,9,10,13,14,18,20 | | 2,3,4,6,7,8,9,13,14,18,20 | |  | |  |
| 7 | 2,3,6,7,8,13 | | 2,3,6,7,8,13 | | 2,3,6,7,8,13 | | *Level II* | |  |
| 8 | 2,3,4,5,6,7,8,9,10,13,14,15,16,17,18,19,20,21 | | 2,3,4,5,6,7,8,9,10,13,14,15,16,17,18,19,20,21 | | 2,3,4,5,6,7,8,9,10,13,14,15,16,17,18,19,20,21 | | *Level II* | |  |
| 9 | 3,4,5,6,7,8,9,10,13,14,15,16,17,20 | | 3,4,5,6,7,8,9,10,13,14,15,16,17,20 | | 4,5,6,8,9,10,13,14,15,16,17,20 | |  | |  |
| 10 | 2,3,4,8,9,10,13,14,18,20 | | 2,3,4,8,9,10,13,14,18,20 | | 8,9,10,13,14,18,20 | |  | |  |
| 13 | 2,3,4,6,7,8,9,10,13,14,15,16,18,19,20 | | 2,3,4,6,7,8,9,10,13,14,15,16,18,19,20 | | 2,3,4,6,7,8,9,10,13,14,15,16,18,19,20 | | *Level II* | |  |
| 14 | 2,3,4,6,7,8,9,10,13,14,18,20 | | 2,3,4,6,7,8,9,10,13,14,18,20 | | 2,3,4,6,8,9,10,13,14,18,20 | |  | |  |
| 15 | 2,3,4,5,6,7,8,9,10,13,14,15,16,17,18,20 | | 2,3,4,5,6,7,8,9,10,13,14,15,16,17,18,20 | | 4,5,8,9,13,15,16,17,18,20 | |  | |  |
| 16 | 2,3,4,5,6,7,8,9,10,13,14,15,16,17,18,20 | | 2,3,4,5,6,7,8,9,10,13,14,15,16,17,18,20 | | 4,5,8,9,13,15,16,17,18,20 | |  | |  |
| 17 | 2,3,4,5,6,7,8,9,10,13,14,15,16,17,18,20 | | 2,3,4,5,6,7,8,9,10,13,14,15,16,17,18,20 | | 4,5,8,9,15,16,17,18,20 | |  | |  |
| 18 | 2,3,4,5,6,7,8,9,10,13,14,15,16,17,18,19,20,21 | | 2,3,4,5,6,7,8,9,10,13,14,15,16,17,18,19,20,21 | | 2,3,6,8,10,13,14,15,16,17,18,19,21 | |  | |  |
| 19 | 2,3,4,5,6,7,8,9,10,13,14,15,16,17,18,19,20,21 | | 2,3,4,5,6,7,8,9,10,13,14,15,16,17,18,19,20,21 | | 8,13,18,19,20,21 | |  | |  |
| 20 | 2,3,4,5,6,7,8,9,10,13,14,15,16,17,19,20,21 | | 2,3,4,5,6,7,8,9,10,13,14,15,16,17,19,20,21 | | 6,8,9,10,13,14,15,16,17,19,20,21 | |  | |  |
| 21 | 2,3,4,6,7,8,13,14,15,16,18,19,20,21 | | 2,3,4,6,7,8,13,14,15,16,18,19,20,21 | | 8,18,19,20,21 | |  | |  |

**Table 3: Iteration 3**

| **Code** | **Reachability Set** | **Antecedent Set** | **Intersection Set** | **Level** |
| --- | --- | --- | --- | --- |
| 4 | 4,5,6,9,14,15,16,17 | 4,5,6,9,14,15,16,17 | 4,5,6,9,14,15,16,17 | *Level III* |
| 5 | 4,5,6,9,14,15,16,17 | 4,5,6,9,14,15,16,17 | 4,5,9,15,16,17 |  |
| 6 | 4,6,9,10,14,18,20 | 4,6,9,10,14,18,20 | 4,6,9,14,18,20 |  |
| 9 | 4,5,6,9,10,14,15,16,17,20 | 4,5,6,9,10,14,15,16,17,20 | 4,5,6,9,10,14,15,16,17,20 | *Level III* |
| 10 | 4,9,10,14,18,20 | 4,9,10,14,18,20 | 9,10,14,18,20 |  |
| 14 | 4,6,9,10,14,18,20 | 4,6,9,10,14,18,20 | 4,6,9,10,14,18,20 | *Level III* |
| 15 | 4,5,6,9,10,14,15,16,17,18,20 | 4,5,6,9,10,14,15,16,17,18,20 | 4,5,9,15,16,17,18,20 |  |
| 16 | 4,5,6,9,10,14,15,16,17,18,20 | 4,5,6,9,10,14,15,16,17,18,20 | 4,5,9,15,16,17,18,20 |  |
| 17 | 4,5,6,9,10,14,15,16,17,18,20 | 4,5,6,9,10,14,15,16,17,18,20 | 4,5,9,15,16,17,18,20 |  |
| 18 | 4,5,6,9,10,14,15,16,17,18,19,20,21 | 4,5,6,9,10,14,15,16,17,18,19,20,21 | 6,10,14,15,16,17,18,19,21 |  |
| 19 | 4,5,6,9,10,14,15,16,17,18,19,20,21 | 4,5,6,9,10,14,15,16,17,18,19,20,21 | 18,19,20,21 |  |
| 20 | 4,5,6,9,10,14,15,16,17,19,20,21 | 4,5,6,9,10,14,15,16,17,19,20,21 | 6,9,10,14,15,16,17,19,20,21 |  |
| 21 | 4,6,14,15,16,18,19,20,21 | 4,6,14,15,16,18,19,20,21 | 18,19,20,21 |  |

**Table 4: Iteration 4**

| **Code** | **Reachability Set** | **Antecedent Set** | **Intersection Set** | **Level** |
| --- | --- | --- | --- | --- |
| 5 | 5,6,15,16,17 | 5,6,15,16,17 | 5,15,16,17 |  |
| 6 | 6,10,18,20 | 6,10,18,20 | 6,18,20 |  |
| 10 | 10,18,20 | 10,18,20 | 10,18,20 | *Level IV* |
| 15 | 5,6,10,15,16,17,18,20 | 5,6,10,15,16,17,18,20 | 5,15,16,17,18,20 |  |
| 16 | 5,6,10,15,16,17,18,20 | 5,6,10,15,16,17,18,20 | 5,15,16,17,18,20 |  |
| 17 | 5,6,10,15,16,17,18,20 | 5,6,10,15,16,17,18,20 | 5,15,16,17,18,20 |  |
| 18 | 5,6,10,15,16,17,18,19,20,21 | 5,6,10,15,16,17,18,19,20,21 | 6,10,15,16,17,18,19,21 |  |
| 19 | 5,6,10,15,16,17,18,19,20,21 | 5,6,10,15,16,17,18,19,20,21 | 18,19,20,21 |  |
| 20 | 5,6,10,15,16,17,19,20,21 | 5,6,10,15,16,17,19,20,21 | 6,10,15,16,17,19,20,21 |  |
| 21 | 6,15,16,18,19,20,21 | 6,15,16,18,19,20,21 | 18,19,20,21 |  |

**Table 5: Iteration 5**

| **Code** | **Reachability Set** | **Antecedent Set** | **Intersection Set** | **Level** |
| --- | --- | --- | --- | --- |
| 5 | 5,6,15,16,17 | 5,6,15,16,17 | 5,15,16,17 |  |
| 6 | 6,18,20 | 6,18,20 | 6,18,20 | *Level V* |
| 15 | 5,6,15,16,17,18,20 | 5,6,15,16,17,18,20 | 5,15,16,17,18,20 |  |
| 16 | 5,6,15,16,17,18,20 | 5,6,15,16,17,18,20 | 5,15,16,17,18,20 |  |
| 17 | 5,6,15,16,17,18,20 | 5,6,15,16,17,18,20 | 5,15,16,17,18,20 |  |
| 18 | 5,6,15,16,17,18,19,20,21 | 5,6,15,16,17,18,19,20,21 | 6,15,16,17,18,19,21 |  |
| 19 | 5,6,15,16,17,18,19,20,21 | 5,6,15,16,17,18,19,20,21 | 18,19,20,21 |  |
| 20 | 5,6,15,16,17,19,20,21 | 5,6,15,16,17,19,20,21 | 6,15,16,17,19,20,21 |  |
| 21 | 6,15,16,18,19,20,21 | 6,15,16,18,19,20,21 | 18,19,20,21 |  |

**Table 6: Iteration 6**

| **Code** | **Reachability Set** | **Antecedent Set** | **Intersection Set** | **Level** |
| --- | --- | --- | --- | --- |
| 5 | 5,15,16,17 | 5,15,16,17 | 5,15,16,17 | *Level VI* |
| 15 | 5,15,16,17,18,20 | 5,15,16,17,18,20 | 5,15,16,17,18,20 | *Level VI* |
| 16 | 5,15,16,17,18,20 | 5,15,16,17,18,20 | 5,15,16,17,18,20 | *Level VI* |
| 17 | 5,15,16,17,18,20 | 5,15,16,17,18,20 | 5,15,16,17,18,20 | *Level VI* |
| 18 | 5,15,16,17,18,19,20,21 | 5,15,16,17,18,19,20,21 | 15,16,17,18,19,21 |  |
| 19 | 5,15,16,17,18,19,20,21 | 5,15,16,17,18,19,20,21 | 18,19,20,21 |  |
| 20 | 5,15,16,17,19,20,21 | 5,15,16,17,19,20,21 | 15,16,17,19,20,21 |  |
| 21 | 15,16,18,19,20,21 | 15,16,18,19,20,21 | 18,19,20,21 |  |

**Table 7: Iteration 7**

| **Code** | **Reachability Set** | **Antecedent Set** | **Intersection Set** | **Level** |
| --- | --- | --- | --- | --- |
| 18 | 18,19,20,21 | 18,19,20,21 | 18,19,21 |  |
| 19 | 18,19,20,21 | 18,19,20,21 | 18,19,20,21 | *Level VII* |
| 20 | 19,20,21 | 19,20,21 | 19,20,21 | *Level VII* |
| 21 | 18,19,20,21 | 18,19,20,21 | 18,19,20,21 | *Level VII* |

**Table 8: Iteration 8**

| **Code** | **Reachability Set** | **Antecedent Set** | **Intersection Set** |  |
| --- | --- | --- | --- | --- |
| 18 | 18 | 18 | 18 | *Level VIII* |
